# Placental trophoblast aging in advanced maternal age is related to increased oxidative damage and decreased YAP

**DOI:** 10.3389/fcell.2025.1479960

**Published:** 2025-01-21

**Authors:** Song Guo, Qihao Pan, Baokang Chen, Yijuan Huang, Si Li, Chenyu Gou, Yu Gao

**Affiliations:** ^1^ Department of Obstetrics and Gynecology, The Sixth Affiliated Hospital of Sun Yat-sen University, Guangzhou, China; ^2^ Biomedical Innovation Center, The Sixth Affiliated Hospital, Sun Yat-sen University, Guangzhou, China

**Keywords:** advanced maternal age, trophoblast aging, YAP, DNA oxidative damage, pregnancy complication

## Abstract

**Introduction:**

The advanced maternal age (AMA) pregnancies escalate rapidly, which are frequently linked to higher risks of adverse outcomes. Advanced maternal age (AMA) placenta exhibited premature aging, presumably resulting in trophoblast dysfunction, inadequate placentation. However, the precise reasons and mechanisms of trophoblast aging in AMA placenta remain unclear, posing a significant limitation to provide effective guidance for prenatal healthcare in clinical settings. Notably, the organism shows heightened vulnerability to oxidative damage as it ages. YAP (Yes-associated protein) was reported to play a critical role in regulation of aging and resisting oxidative damage, yet these roles had not been elucidated in the placenta. Therefore, this study explored the relationship between trophoblast cell aging and oxidative injury and YAP in AMA pregnancy, which not only provided an insight into the mechanisms of trophoblast cell aging, but also provide valuable directions for healthcare during AMA pregnancy.

**Methods:**

In this study, human term placentas were collected from AMA and normal pregnancies for the analysis of aging, oxidative damage and YAP level. HTR8/SVneo cells were manipulated with (hydrogen peroxide) H_2_O_2_ to explore the effects of oxidative damage on trophoblast cell senescence and YAP levels. YAP expression in HTR8/SVneo cells was manipulated to investigate its role in trophoblastic senescence and oxidative damage.

**Results:**

Compared with the control group, the AMA placenta exhibits increased aging biomarkers, which is coupled with an elevation in oxidative damage within placental trophoblast cells and a notable decline in YAP levels. Cellular experiments demonstrated that oxidative damage from H_2_O_2_ triggered trophoblast cell senescence and resulted in a reduction of YAP levels. Furthermore, employing molecular modification to silence YAP expression in these cells led to an induction of aging. Conversely, overexpressing YAP ameliorated both trophoblast cell aging and the associated DNA oxidative damage that arised from H_2_O_2_.

**Conclusion:**

The decline of YAP in AMA pregnancy should be responsible for the increased oxidative injury and premature placenta aging, indicating that YAP plays a significant role in combating oxidative damage and delaying aging, thereby providing a new guidance for prenatal care in AMA pregnancies. Maintaining YAP levels or implementing anti-oxidative stress interventions could potentially mitigate the incidence of complications involved AMA pregnancy.

## Introduction

A growing number of women have pregnancies at an advanced maternal age (AMA), typically defined as 35 years or older (Cooke and andDavidge, 2019), which is associated with numerous pregnancy complications (Marozio et al., 2019; Norwitz, 2007), such as preeclampsia (PE), severe intrauterine growth restriction (IUGR), miscarriage, and stillbirth. Accumulative evidence suggests that these adverse pregnancy outcomes are implicated in aberrant placental aging of AMA (Xiong et al., 2021; Chen et al., 2021). Placenta aging is characterized with progressive decrease of function at the cellular, tissue, and organ level, contributing to a reduced adaptability to stress and an increased vulnerability to disease and mortality (Fedarko, 2011).

Pregnancy is a state of increased oxidative stress (Belo et al., 2004;Burton and and Jauniaux, 2004), which particularly focuses in the human placenta supra physiologically. The AMA placentas show more fragile response to oxidative stress than those young (Liao et al., 2019; Pomatto and and Davies, 2018). As a common inducer of aging, oxidative stress could lead to a series of events, including damnification to cellular lipids, proteins, DNA and ultimately result in aging (Hernandez-Segura et al., 2017). Oxidative stress of the gestation is of great correlation with the pathomechanism of adverse pregnancy outcomes. More precisely, placental trophoblasts from pregnancies complicated by abortion, PE and IUGR exhibit higher levels of oxidative stress and aging biomarkers than those from normal pregnancies (Huppertz, 2008; Sultana et al., 2017). And AMA is a high-risk factor of these complications.

Aging is an aftereffect of the interaction between environmental and genetic factors, and some genes play a vital role in regulating aging, including Yes-associated protein (YAP), which was originally identified as a key transcription factor in Hippo pathway of controlling organ size, tissue regeneration, cell fate decisions (Russell and Camargo, 2022). In recent years, YAP as the main effector of cellular mechanical signals, its attenuated function was reported linked to the decline of structure and function in aging tissues and organs (Sladitschek-Martens et al., 2022), implying that YAP may participate in regulation of senescence and resisting oxidative damage. However, the role of YAP in placenta aging and oxidative damage remained unknown in the AMA pregnancies, while it was reported the reduction of YAP in Series of pregnancy complications (Liao et al., 2022). In the present study, we aimed to explore the relationship among aging, oxidative damage and YAP in placenta trophoblast, providing valuable directions for healthcare during AMA pregnancy.

## Materials and methods

### Patient and specimen collection

The term placenta tissues were collected from surgical terminations with approval of the Sixth Affiliated Hospital of Sun Yat-sen University Committee on the Ethics of Human Research (No: L2022ZSLYEC-014) and in accordance with the principles outlined in the Declaration of Helsinki. All samples were collected with the patients’ written informed consent. Young pregnancies (25–30 years old, 37.57–40.71 weeks, n = 23) and AMA pregnancies (35–45 years old, 37–40.43 weeks, n = 19) samples were collected within 15 min after delivery. Patients with major pregnancy complications, such as IUGR, PE, or gestational diabetes mellitus (GDM), were excluded. Part of the samples underwent PBS washing, followed by immediate freezing in liquid nitrogen and storage at −80°C until further processing. Another part of the samples was fixed overnight in 4% paraformaldehyde and subsequently embedded in paraffin or OCT compound (Servicebio, Wuhan, China) for future applications. The clinical characteristics of the pregnant women are outlined in Table 1.

**TABLE 1:** Clinical characteristics of the patients who provided term placental tissue.

Parameters	Control (n = 23)	Advanced maternal age (AMA) (n = 19)
Age (years)	27.61 ± 2.06	37.89 ± 3.30****
Gestational age at delivery (weeks)	39.11 ± 1.13	38.35 ± 0.88
Body mass index (BMI; kg/m^2^)	25.96 ± 2.70	27.41 ± 2.86
Gravidity	1.39 ± 0.50	2.05 ± 1.02
Systolic blood pressure (mmHg)	113.9 ± 6.93	116.6 ± 6.77
Diastolic blood pressure (mmHg)	71.5 ± 4.10	72.9 ± 4.21
Weight of placenta (g)	491.30 ± 32.52	467.89 ± 73.60
Weight of neonatus (g)	3204.35 ± 437.65	2983.16 ± 529.81

Data are expressed as the mean ± SEM. The data were analyzed by Student’s t-test. ****P < 0.0001.

### Cell culture

The immortalized human trophoblast cell line HTR8/SVneo, obtained from the American Type Culture Collection (ATCC, Inc., Manassas, USA), was cultured in Roswell Park Memorial Institute medium (RPMI) 1,640 supplemented with 10% FBS (FSP500, ExCell Bio, Suzhou, China) and 1% penicillin-streptomycin (Cod. 15,140,122, Gibco, Waltham, United States). The cells were maintained under standard culture conditions (37°C and 5% CO2 in a humidified environment). Various concentrations of H_2_O_2_ were prepared by diluting 3% H_2_O_2_ (Cod. 323381-25ML, Sigma-Aldrich, St. Louis, United States) with RPMI medium.

### Cell transfection

Briefly, the HTR8/SVneo cells were seeded into 6-well plate and maintained overnight, and then transiently transfected with indicated plasmid when cells were 50%–70% confluent. After infection for 24 h, cells were collected for detected the efficiency of transfection by Western blotting (WB). The short-hairpin RNA plasmid targeting YAP and the YAP-overexpression plasmid were obtained from IGEbio, Guangzhou, China. The target sequences for sh-YAP was: GCC​ACC​AAG​CTA​GAT​AAA​GAA. The sequences for the YAP overexpression are listed in Supplementary Figure S1. The YAP-knockdown and YAP-overexpression plasmids were constructed by IGEbio, China, through the plasmid vectors PLKO-puro and Plvx-puro respectively, with corresponding empty plasmid vectors as control. The detailed methods and reagents could be found in the Supplementary Materials.

### Cell viability assay

The impact of H_2_O_2_ on the viability of HTR8/SVneo cells was assessed using a CCK8 Assay kit (Cod. 1,018, APExBIO, Huston, United States). Cells were seeded at a density of 10^4^ cells per well in 96-well plates. The following day, the cells were exposed to varying concentrations of H_2_O_2_ (0, 15, 37.5, 75, 100, 150, 200 μM). After being treated with H_2_O_2_ for durations of 0.5, 1, 4, 24, and 48 h, the liquid above the sediment was aspirated. Subsequently, CCK8 reagent was introduced into every well and allowed to incubate for 2 h. The absorbance reading at 450 nm was measured using a scanning multiwell spectrophotometer (Thermo Fisher Scientific, Waltham, United States). The cell viability was calculated using the following formula: viability (%) = [(As-Ab)/(Ac-Ab)] ×100 (As = absorbance of the experimental well, Ab = absorbance of the blank well, Ac = absorbance of the control well).

### H_2_O_2_ treatment in HTR8/SVneo cells

To manufacture oxidative stress in HTR8/SVneo cells, the complete medium was replaced with medium containing H_2_O_2_ at the IC50 concentration of 25 µM for 30 min, when the cells reached 60%–70% confluence. After 30 min, the medium containing H_2_O_2_ was replaced with normal complete medium for continued culturing. Thereafter, ensure fresh medium is exchanged every 2 days. Cells are harvested at designated time after H_2_O_2_ exposure (0.5 h, 24 h, 48 h, 72 h, 96 h, 120 h, 144 h) for subsequent experiments, such as total protein extraction, senescence-associated β-galactosidase staining.

### Senescence-associated β-galactosidase staining (SA-β-Gal staining)

The SA-β-Gal staining was performed on frozen sections of tissues and HTR8/SVneo cells in 6-well plates using a commercial kit (Cod. C0602, Beyotime, Shanghai, China) according to the manufacturer’s instructions. Briefly, frozen tissue sections and trophoblast cells were fixed for 15 min with 3.7% formaldehyde at room temperature and then washed 3 times with PBS and stained overnight at 37°C in a CO2-free atmosphere in nonhumidified incubator. After incubation, the cells and tissue sections were visualized using light microscopy (Olympus, Tokyo, Japan), and images were captured. Blue signals were treated as positive signals, and the positive rate were calculated in 3 random-view fields of each sample in 3 independent experiments.

### Western blotting (WB)

Proteins were extracted entirely by utilizing RIPA lysis buffer (Cod. P0013B, Beyotime, Shanghai, China), supplemented with 1% cocktail of phosphatase inhibitor and 1% cocktail of protease inhibitor. Concentrations were quantified using a BCA protein assay kit (Cod. P0010, Beyotime, Shanghai, China). The lysates were combined with 5x SDS-PAGE sample buffer, boiled for 10 min, and then allowed to cool to room temperature. Equal quantities of protein were loaded onto SDS-PAGE gels (Bio-Rad, California, United States), followed by transfer onto PVDF membranes (Sigma-Aldrich, St. Louis, United States) for immunoblot analysis. Membranes were blocked for 1 h at room temperature (RT) in 5% evaporated milk diluted in Tris-buffered saline (TBS) and 0.1% Tween 20 and incubated with the following primary antibodies overnight at 4°C: anti-YAP (1: 1000, Cod. 14074S, Cell Signaling, Danvers, United States), anti-p53 (1: 1000, Cod. sc-126, Santa Cruz, Dallas, United States), anti-p21 (1: 1000, Cod. 2,947, Cell Signaling, Danvers, United States), anti-p16 (1: 1000, Cod. sc-166760, Santa Cruz, Dallas, United States), anti-β-actin (1:1000, Cod. GB15001-100, Servicebio, Wuhan, China), anti-GAPDH (1:1000, Cod. GB15002-100, Servicebio, Wuhan, China). After washing and incubating with the secondary antibodies (1: 10,000, Cod. K1221 and K1223, APExBIO, Huston, United States) for 1 h at RT, immunoreactive proteins were visualized by the ECL (Cod. BL520A, Biosharp, Hefei, China) plus chemiluminescence system (Bio-Rad, California, United States) following the manufacturer’s instructions. Protein bands were quantified using ImageJ software.

### Apoptosis detection

Apoptosis was conducted as previously described (Pan et al., 2023). Briefly, HTR8/SVneo cells from distinct groups were collected, including those in cultured media, and incubated with Annexin V/PI (Cod. E-CK-A211, Elabscience, Wuhan, China) for 15 min at RT in the absence of light according to the manufacturer’s instructions. The labeled cells were then analyzed utilizing a flow cytometry (BECKMAN, Brea, United States).

### Immunohistochemistry (IHC)

The placental tissues were fixed overnight with 4% paraformaldehyde at RT, followed by dehydration and embedding in paraffin. Subsequently, the tissues were sectioned into 4-μm-thick slices. In preparation for IHC analysis, the tissue sections underwent deparaffinization and rehydration through a series of graded alcohol solutions. Next, the sections were subjected to antigen retrieval by boiling in a pressure cooker containing Tris-EDTA (pH 9.0) for 10 min. Following this step, the sections were allowed to cool down to RT naturally and treated with 3% H_2_O_2_ for 15 min to suppress endogenous peroxidase activity. Next, the sections were incubated with mouse mAb against p53 (1:50, Cod. sc-126, Santa Cruz, Dallas, United States), rabbit mAb against p21 (1:400, Cod. 2,947, Cell Signaling, Danvers, United States), mouse mAb against p16 (1:50, Cod. sc-166760, Santa Cruz, Dallas, United States), mouse mAb against 8-OHdG (1:50, Cod. sc-393871, Santa Cruz, Dallas, United States) and PBS for negative control at 4°C overnight, followed by treatment with the secondary antibody (1: 200, Cod. K1221 and K1223, APExBIO, Huston, United States) conjugated with horseradish peroxidase for 30 min at RT. The immunocomplexes were then visualized with diaminobenzidine. The images were captured under a light microscope (Olympus, Tokyo, Japan). Each slide was visualized and several images (3–5 per placenta) were captured per placenta. The positive staining was determined using ImageJ. Briefly, we quantified three random-view fields for each sample. The percentage of positively stained trophoblast nuclei was calculated relative to the total number of trophoblast nuclei counted.

### Immunofluorescence (IF)

Immunostaining was performed on HTR8/SVneo cells. Briefly, the cells were washed by PBS and fixed with 4% paraformaldehyde at RT for 15 min. Then they were permeabilized with 0.3% Triton X-100 and blocked with 5% bovine serum albumin (BSA) (Beyotime, Wuhan, China). After that, the cells were incubated with primary antibodies against 8-OHdG (1:50, Cod. sc-393871, Santa Cruz, Dallas, United States) at 4°C overnight, followed by incubation with Alexa-Fluor 488 (1:500, Cod. 34,963, Cell Signaling, Danvers, United States) or Cy3-labeled (1:200, Cod. GB21303, Servicebio, Wuhan, China) secondary antibodies for 1 h at room temperature in darkness. The nuclei were stained with 4′,6-diamidino-2-phenylindole (DAPI) for 5 min, and images were visualized using a confocal microscopy (Leica, Wetzlar, Germany).

### Matrigel invasion assay

The invasion assay was conducted using a 24-well plate with membrane inserts (Corning, New York, United States) equipped with 8-mm-pore-sized polycarbonate filters that were pre-coated with 50 μL of Matrigel matrix (Cod. 356,234, Corning, New York, United States) solution at a concentration of 1 mg/mL 300 μL of serum-free culture medium containing 8 × 10^4^ cells was added to each insert, which was then positioned in the lower chamber filled with 600 μL of culture medium containing 10% FBS. Following a 24-hour incubation period, the cells that migrated through the membrane were fixed using 4% paraformaldehyde and stained with crystal violet. Subsequently, images were taken using light microscopy (Olympus, Tokyo, Japan), and the data were analyzed using ImageJ software (Fiji, NIH, Bethesda, United States).

### Wound-healing assay

HTR8/SVneo cells were plated in 6-well plates and cultured until reaching over 90% confluence, and then a scratch on the cell monolayer was introduced using a pipette tip. The cells were then rinsed 3 times with fresh culture medium and cultured for another 24 h. Pictures were taken at 0 and 24 h post-scratch. The area of wound healing was quantified with ImageJ software. Experiments were repeated in triplicate.

### RNA sequencing

Negative control and YAP-knockdown HTR8/SVneo cells were collected for total RNA extraction using TRIzol reagent (Invitrogen, Carlsbad, United States). Then RNA quality was determined by 5,300 Bioanalyser (Agilent) and quantified using the ND-2000 (NanoDrop Technologies, San Diego, United States). RNA purification, reverse transcription, library construction and sequencing were performed at Shanghai Majorbio Bio-pharm Biotechnology Co. Ltd. (Shanghai, China) according to the manufacturer’s instructions (Illumina, San Diego, CA). A total of 1 μg of RNA from each sample served as input material for the sample preparations. Essentially, differential expression analysis was performed using the DESeq2. Differentially expressed genes (DEGs) with |log2FC| ≧ 1 and padj ≤ 0.05 were considered to be significant. In addition, functional-enrichment analysis were performed using GESA analysis to identify which DEGs were significantly enriched.

### Statistical analysis

Data are presented as the mean ± SEM. Statistical analysis was conducted using GraphPad Prism 8 software (La Jolla, Wilmington, USA) by Student's t-test or one-way ANOVA. Asterisks represent the following p values: *p < 0.05, **p < 0.01, ***p < 0.001, ****p < 0.0001, and ns, no significant.

## Results

### AMA placentas showed a aging phenotype and were associated with decreased YAP and increased DNA oxidative damage

**FIGURE 1:**
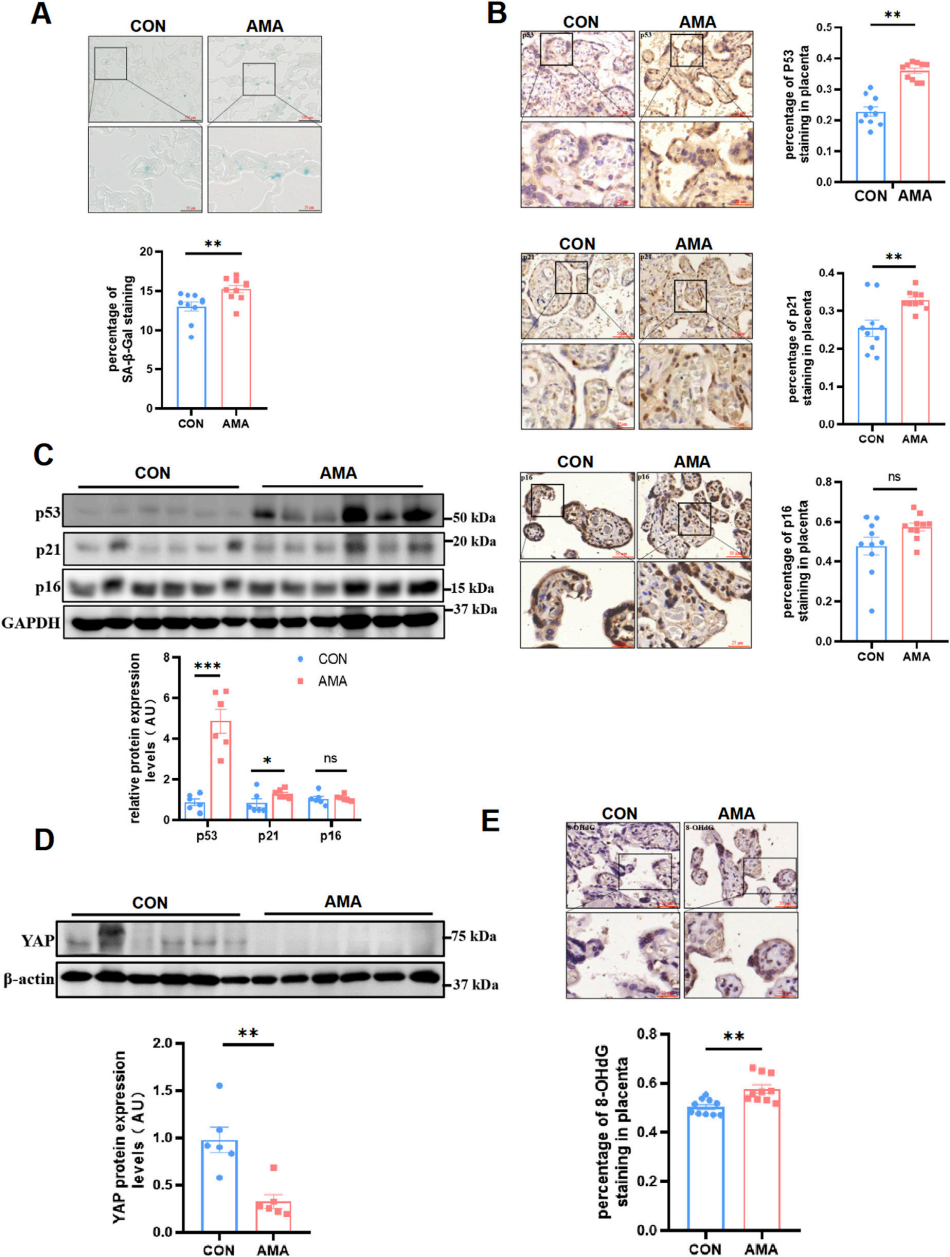
AMA placentas showed a aging phenotype and were associated with decreased YAP and increased DNA oxidative damage. **(A)** SA-β-Gal staining of human term placenta sections (n = 10 patients per group, 3 random fields per patient). Scale bars, 100 μm. **(B)** IHC staining of p53, p21 and p16 in human term placentas (n = 10, 3 fields per patient). Scale bars, 50 μm. **(C)** Western blotting of p53, p21 and p16 protein expression in human term placentas (n = 6). **(D)** Western blotting of YAP protein expression in human term placentas (n = 6). **(E)** IHC staining of 8-OHdG in human term placentas (n = 10, 3 fields per patient). All data are presented as the mean ± SEM. *p < 0.05, **p < 0.01. Student’s t test. AU, arbitrary unit.

Human term placentas from AMA pregnancies showed more positive senescence-associated β-galactosidase (SA-β-Gal) staining than those from young pregnancies (Figure 1A). In accordance with this result, multiple well-known biomarkers of senescence were also observed increased in AMA term placentas, including p53 and p21, through IHC (Figure 1B) and Western blot (Figure 1C) detection. Yet, another familiar senescence biomarker p16 displayed less significant difference between the AMA and young pregnancies (Figures 1B, C). They are related to the cell cycle, not only express in placental trophoblast cells but also in villous stromal cells. In a word, our results confirmed that AMA placentas were of more severe senescence. While it was reported that YAP level apparently decreased in organs with age, whether AMA placentas had similar manifestation remained unknown. Then we analyzed YAP protein level in human placentas and found that YAP expression was significantly downregulated in AMA placentas, as demonstrated by Western blot (Figure 1D). In addition, we were aware that aging was of great association with DNA damage and the oxidative stress, which accumulated as people got old. 8-hydroxy-2′-deoxyguanosine (8-OHdG), an oxidized derivative of deoxyguanosine, is the predominant form of oxidative DNA lesions and thus was widely used to represent the oxidative DNA damage (Londero et al., 2016). Therefore, we measured the level of 8-OHdG in term placentas. The findings showed increased percentage of immunopositive nuclei in AMA group compared to gestation-matched controls (Figure 1E), indicating a higher level of oxidative DNA damage. Taken together, our findings suggested that the AMA placentas were related with intensified cellular senescence, which was accompanied with the deficiency of YAP and heighten of oxidative damage.

### Oxidative stress induced senescence and attenuated YAP expression in HTR8/SVneo cells

**FIGURE 2:**
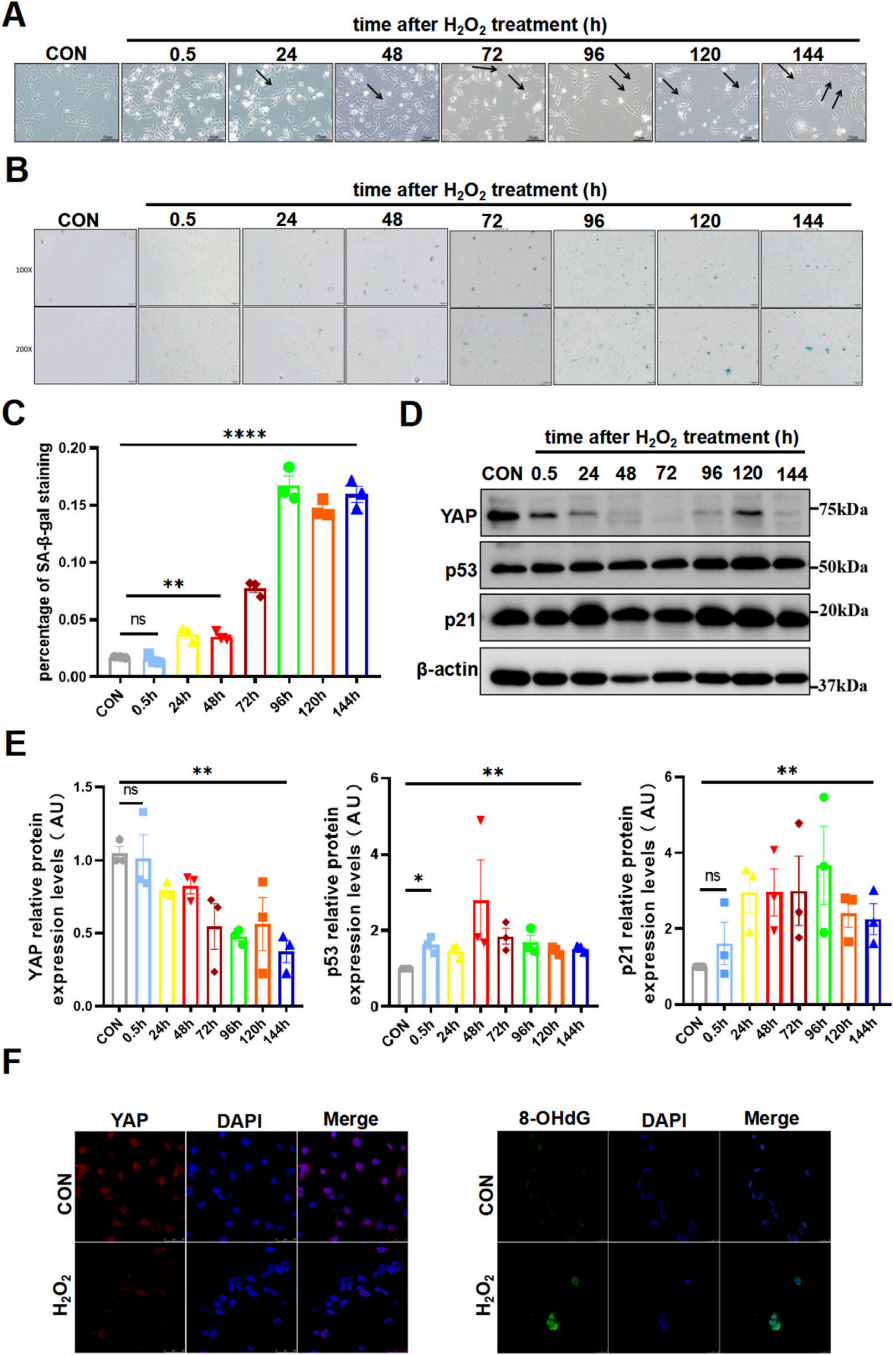
Oxidative stress induced senescence and attenuated YAP Expression in HTR8/SVneo cells. **(A)** HTR8/SVneo cells morphology changed over time by light microscopy after treated with H_2_O_2_ (n = 3). The arrow indicated the cells that have become larger and flattened in morphology, which was the characteristic of senescence. **(B)** SA-β-Gal staining in HTR8/SVneo cells by light microscopy over time after exposure of H_2_O_2_ (n = 3). Scale bars, 50 μm. **(C)** Statistical analysis diagram of the SA-β-Gal staining results in **(B)** (n = 3). **(D)** Western blotting of YAP, p53, and p21 protein expression in HTR8/SVneo cells over time after treated with H_2_O_2_ (n = 3). **(E)** Semi-quantitative statistical analysis diagram of various proteins of **(D)** (n = 3). **(F)** IF staining of YAP (red) and 8-OHdG (green) in different HTR8/SVneo cells; nuclei were counterstained with DAPI (blue). Scale bars, 100 μm (n = 3). All data are presented as the mean ± SEM. *p < 0.05, **p < 0.01, and ns, no significant. Student’s t-test for two groups, and one-way ANOVA for three or more groups. ns, nonsignificant; AU, arbitrary unit. All data are representative of three independent experiments.

To further investigate the regulations among aging, YAP and oxidative damage in trophoblast cells, we selected HTR8/SVneo cell line for subsequent experiments and engendered oxidative damage with the common oxidizing agent H_2_O_2_. As has been previously reported, H_2_O_2_ exerted a toxic effect on HTR8/SVneo cells in a time-dose-dependency manner by CCK-8 assay (Supplementary Figure S2), which showed an IC50 of 25 µM for treatment of 30 min. Since it typically took some time before cells developed senescent after a destructive stimulus, we then observed the cell changed over consecutive days. The stimulated cells gradually got abnormally enlarged and flattened morphology (Figure 2A), which was a peculiarity of senescent cells. Simultaneously, positive SA-β-Gal staining in the cells became increasingly obvious from the third day after the H_2_O_2_ attack (Figures 2B, C). Furthermore, the expression of p53 and p21 rose up rapidly after H_2_O_2_ stimulation and kept relatively high level for days (Figures 2D, E). Yet, another cyclin-dependent kinase inhibitor p16 did not display specific trend among groups (Supplementary Figure S3). Interestingly, YAP was diminished and remained low following oxidative damage (Figures 2D, E). And consistent with our hypothesis, the oxidised DNA increased notably, as represented by enhanced fluorescence intensity of 8-OHdG (Figure 2F). These findings indicated that oxidative stress contributed to senescence, along with a reduction of YAP expression and promotion of DNA damage.

### YAP deficiency resulted in senescence and DNA oxidative damage in HTR8/SVneo cells, and compromised its invasiveness

**FIGURE 3:**
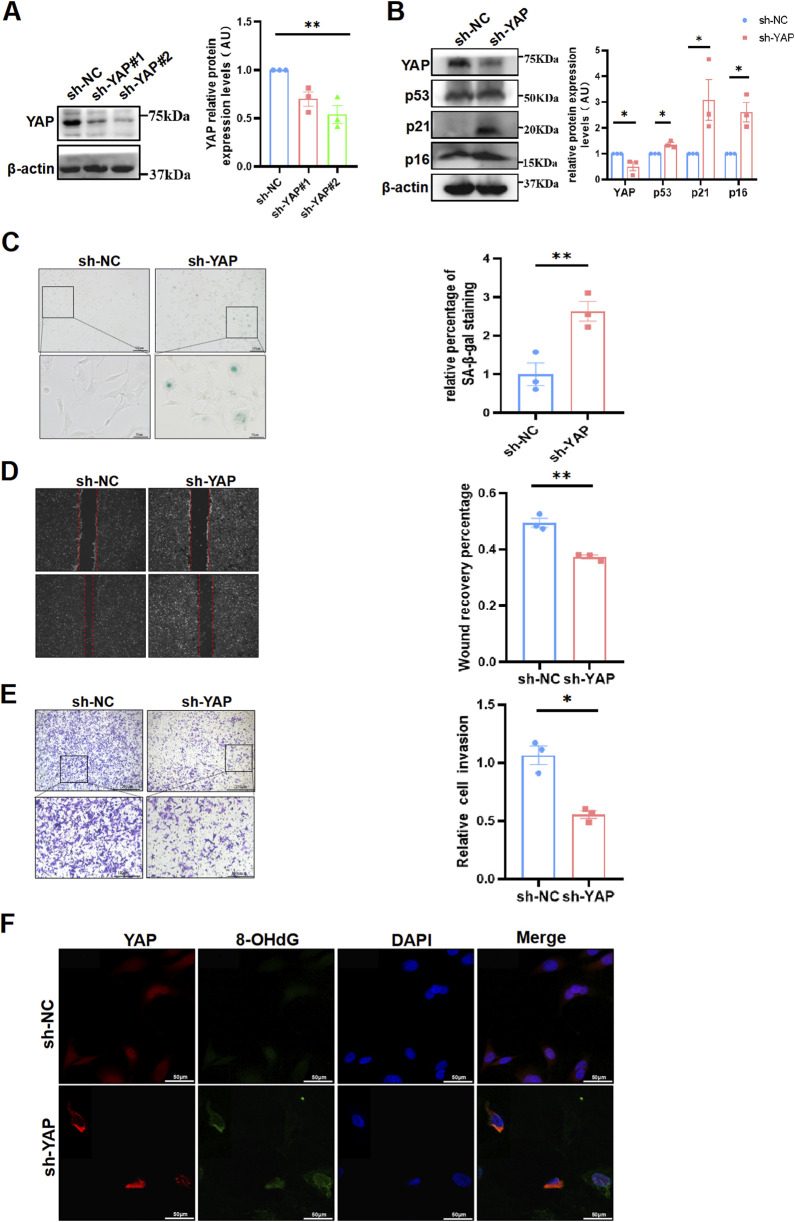
YAP deficiency resulted in senescence and DNA oxidative damage in HTR8/SVneo cells, and compromised its invasiveness. **(A)** Western blotting of YAP expression in different HTR8/SVneo cells. sh-NC, negative control cells transfected with scramble shRNA; sh-YAP, cells transfected with shRNAs targeting YAP (n = 3). **(B)** Western blotting of YAP, p53, p21 and p16 expression in different HTR8/SVneo cells. Sh-YAP#1 was used in the following experiments (n = 3). **(C)** Representative SA-β-Gal staining in various HTR8/SVneo cells (n = 3). Scale bars, 100 μm. **(D)** Wound-healing assay of HTR8/SVneo cells (n = 3). Scale bars, 100 μm. **(E)** Transwell invasion assay of HTR8/SVneo cells (n = 3). Scale bars, 250 μm. **(F)** IF staining of YAP (red) and 8-OHdG (green) in HTR8/SVneo cells; nuclei were counterstained with DAPI (blue) (n = 3). Scale bar, 100 μm. All data are presented as the mean ± SEM. *p < 0.05, **p < 0.01, ***p < 0.001. Student's t test. AU, arbitrary unit. All data are representative of three independent experiments.

To examine whether YAP is a determinant of aging in placental trophoblasts, we introduced shRNA-mediated YAP knockdown in HTR8/SVneo cells (sh-YAP) and validated the efficiency by Western blot (Figure 3A). As a result, we observed an increase in the expression of senescent biomarkers, such as p53, p21 and p16 through WB (Figure 3B). In line with these observations, sh-YAP cells also demonstrated higher levels of SA-β-Gal staining compared to the controls (Figure 3C). Moreover, to evaluate the impact of YAP on trophoblast functions, HTR8/SVneo cells with manipulated YAP expression were subjected to migration and invasion assays. It was shown in the wound-healing assay, there was significant repression of migratory capability in sh-YAP cells (Figure 3D). Consistently, the results of the Matrigel transwell assay demonstrated that ablation of YAP resulted in a marked suppression of invasiveness in HTR8/SVneo cells (Figure 3E), while apoptosis rates of cells did not differ among the various groups (Supplementary Figure S4). Subsequently, we conducted immunofluorescence staining to detected the 8-OHdG in these cells, which showed an increase in sh-YAP cells compared to the controls (Figure 3F). These facts confirmed that YAP deficiency in trophoblasts induced senescence and was responsible for increased DNA damage.

### Overexpression of YAP ameliorated senescence and DNA oxidative damage induced by H_2_O_2_ in HTR8/SVneo cells

**FIGURE 4:**
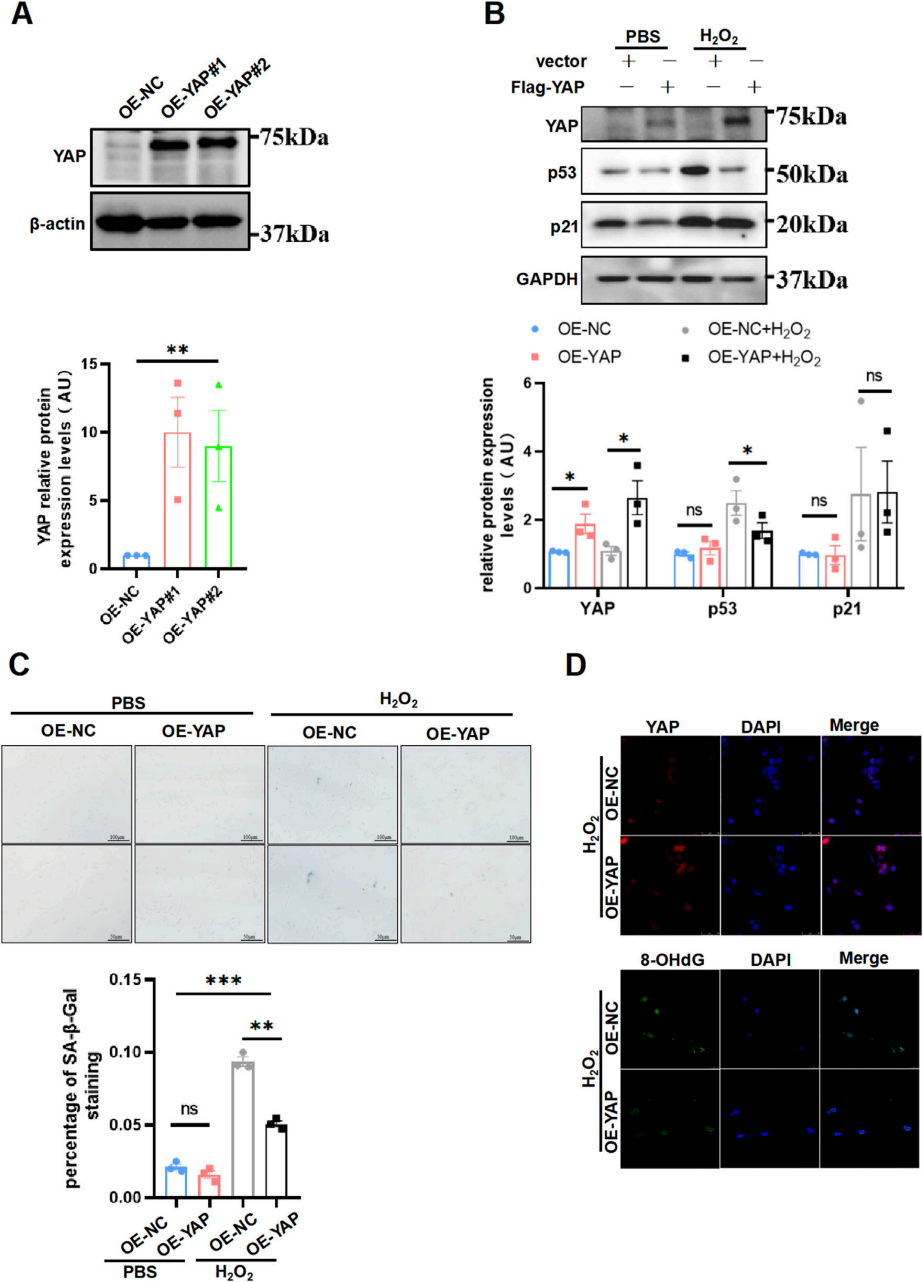
Supplement of YAP ameliorated aging and DNA oxidative damage induced by H_2_O_2_ in HTR8/SVneo cells. **(A)** Western blotting of YAP expression in different HTR8/SVneo cells (n = 3). OE-NC, negative control; OE-YAP, YAP overexpression. **(B)** Western blotting of YAP, p53, p21, and p16 expression in different HTR8/SVneo cells. OE-YAP#1 was used in the following experiments (n = 3). **(C)** Representative SA-β-Gal staining in various HTR8/SVneo cells (n = 3). Scale bars, 100 μm. OE-NC + H_2_O_2_, negative control cells treated with H_2_O_2_; OE-YAP + H_2_O_2_, YAP overexpression cells treated with H_2_O_2_. **(D)** IF staining of YAP (red) and 8-OHdG (green) in HTR8/SVneo cells; nuclei were counterstained with DAPI (blue) (n = 3). Scale bar, 100 μm. All data are presented as the mean ± SEM. *p < 0.05, **p < 0.01, ***p < 0.001, and ns, nonsignificant. Student’s t-test for two groups, and one-way ANOVA for three or more groups. AU, arbitrary unit. All data are representative of three independent experiments.

Now that loss of YAP leading to cell senescence, whether augmentation of YAP could retroact aroused plenty interest to us. To corroborate this idea, first we generated the YAP-overexpression HTR8/SVneo cells (Figure 4A). Next, the cells were exposed to H_2_O_2_ using the same concentration and duration as in previous experiments for inducing oxidative damage, after which the cells were performed a senescent staining to evaluate the aging phenotype. We found that p53 expression was decreased, whereas p21 expression did not differ between the groups (Figure 4B). Additionally, the YAP-overexpression HTR8/SVneo cells exhibited less SA-β-Gal staining than the NC group (Figure 4C). Accordingly, YAP-overexpression cells exhibited a weaker staining intensity of 8-OHdG compared to controls by immunofluorescence staining (Figure 4D). However, exclusive overexpression of YAP did not have any impact on 8-OHdG under no oxidative damage (Supplementary Figure S5). Collectively, these results implied that overexpressing YAP could compromised senescence induced by H_2_O_2_ in placental trophoblast, potentially due to a protection effect in reducing DNA oxidative damage.

### The transcriptome was altered in YAP-knockdown HTR8/SVneo cells

**FIGURE 5:**
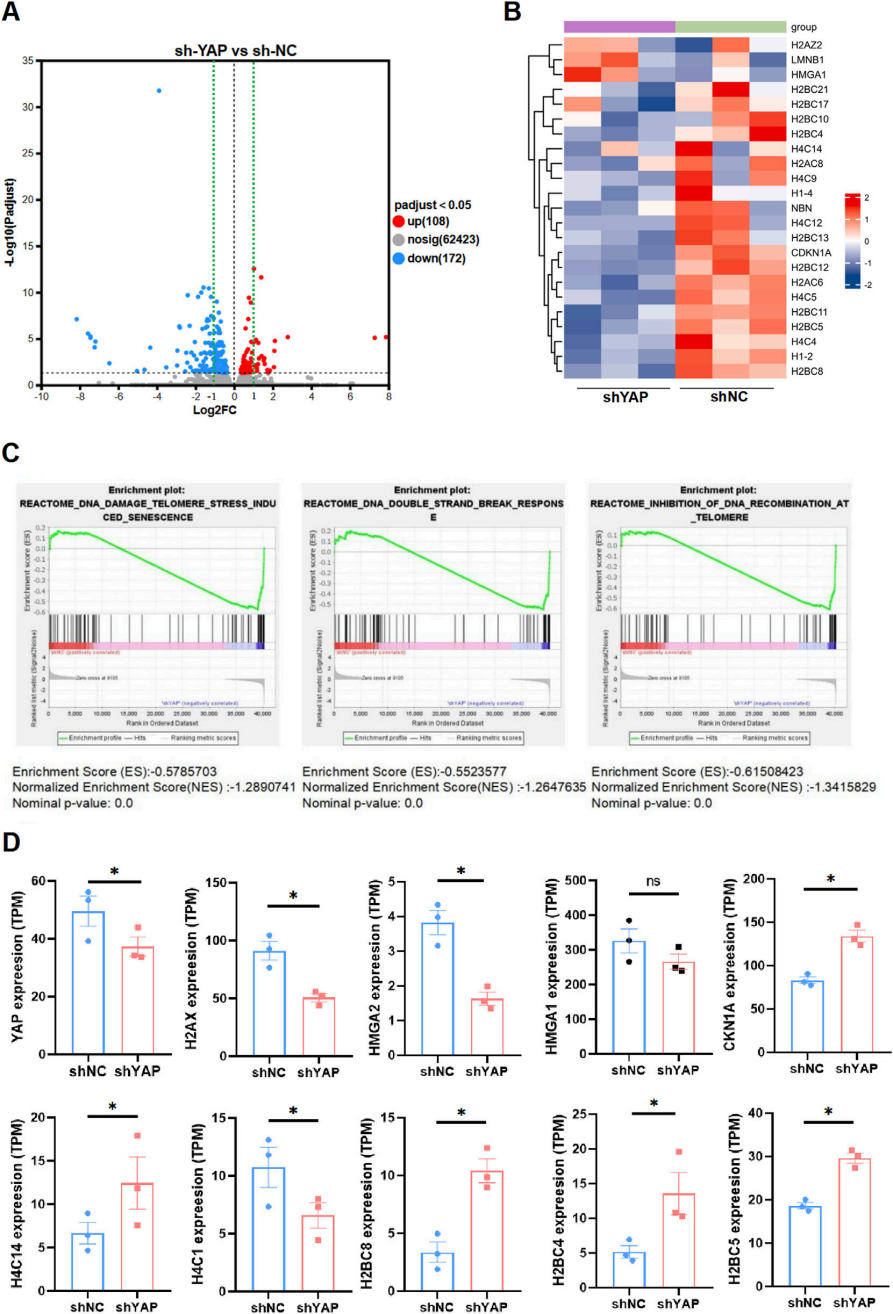
The transcriptome was altered in YAP-knockdown HTR8/SVneo cells. **(A)** Volcano plot of the significant differences in gene expression levels between sh-YAP and sh-NC HTR8/SVneo cells. sh-NC, negative control cells transfected with scramble shRNA; sh-YAP, cells transfected with shRNAs targeting YAP#1 (padj <0.05, n = 3). **(B)** Heat map of differentially expressed genes (DEGs) between sh-YAP and sh-NC by GESA analysis (padj <0.05, n = 3). **(C)** Multiple DNA damage related pathways were enriched including the DNA damage telomere stress induced senescence, DNA double-strand break pathways and inhibition of DNA recombination at telomere. **(D)** Further investigation of our RNA-seq data revealed that many genes involved in theses pathways were significantly changed in sh-YAP. *p < 0.05, and ns, nonsignificant. Student's t test.

To explore the potential mechanism underlying YAP in senescence regulation and alleviating oxidative damage, sh-YAP and sh-NC HTR8/SVneo cells were subjected to mRNA sequencing, revealing substantial differences in gene expression. Specifically, sh-YAP cells exhibited significant variations in the expression levels of 280 mRNAs (108 upregulated and 172 downregulated) in comparison to sh-NC (Figure 5A). Then we conducted GSEA analysis on our RNA-seq data. The results showed differentially expressed genes (DEGs) between sh-NC and sh-YAP HTR8/SVneo cells (Figure 5B) and multiple DNA damage related pathways were enriched, including the DNA damage telomere stress induced senescence, DNA double-strand break pathways and inhibition of DNA recombination at telomere (Figure 5C). Notably, many of these DEGs are related to histone expression, which plays crucial roles in transcriptional regulation, DNA repair, DNA replication, and chromosomal stability according to National Center for Biotechnology Information (NCBI). Further investigation of our RNA-seq data revealed that many genes involved in theses pathways were significantly changed in sh-YAP group (Figure 5D). These data suggest that downregulation of YAP triggers changes in numerous histone genes, involving signaling pathways such as stress-induced DNA damage.

## Discussion

Growing number of evidence has shown that pregnancy complications are inclined to happen in AMA pregnancies compared with the young. These adverse outcomes of AMA pregnancy were connected to placenta of aging (Woods et al., 2017; Li et al., 2017). Cellular senescence is an inevitable experience in the life cycle of all living organisms, which results from progressive DNA damage. An obvious characteristic of aging is gradual decline in physiological functions occurring at the cellular, tissue, and organic level but more susceptible to disease and mortality, which is compatible with a high rate of pregnancy complications in AMA pregnancy. In senescent cells and tissues, the senescence-associated β-galactosidase (SA-β-Gal) has increased activity and is the most common and representative biomarker. In addition, increased levels of p53, p21 have been also involved with senescence and considered to be important biomarkers. The present study revealed these biomarkers above were of higher level in the human AMA placenta, which is consistent with previous results (Xiong et al., 2021; Chen et al., 2021). That was to say, AMA placenta has an apparent senescent phenotype.

p53, p21, and p16, which are related to the cell cycle, all express in placental trophoblast cells and villous stromal cells. p21 can act as a downstream of p53, and they consistently mediate irreversible DNA damage, contributing to the stability and maintenance of cellular senescence, potentially without increasing p16 (De Oliveira, 2006). During the physiological aging of placenta, gradual rise in p53, p21, and p16 (Cindrova-Davies et al., 2018) suggested that they may be necessary for the maturation of placenta. These senescence markers may facilitate the secretion of senescence-associated secretory phenotypes (SASP), promoting the initiation of labor. Unlike the programmed senescence characteristics observed in embryos, abnormal senescence in placenta shared common features with DNA damage-induced senescence and exhibited coordinated activation of the p53-p21 regulatory pathway (Chuprin et al., 2013). In placenta with PE or post-term pregnancy, premature increase in p53 and p21 may lead to placental dysfunction, resulting in adverse pregnancy outcomes (Cox and Redman, 2017; Sultana et al., 2018), but in these PE placentas, p16 showed no statistically significant increase (Cox and Redman, 2017), which was consistent with our observations in AMA placenta, suggesting that pathological placental senescence may be induced in a p16-independent manner. In fact, cellular senescence can be induced in a p53-or p16-independent way (De Oliveira, 2006; Ho et al., 2011). However, it remains plausible that p16 accumulates swiftly during placental senescence, maintaining elevated levels in late-gestation placenta, potentially rendering rise in p16 less discernible in pathological senescence. p53, p21, and p16 occupy pivotal positions and serve crucial functions within placental tissue. They collectively sustain normal proliferation, differentiation, and apoptosis processes in placental cells through intricate regulatory frameworks, guaranteeing optimal placental development and functionality.

YAP, previously known as the chief effector of cellular mechanosignalling, was reported to be involved in regulation aging independent of Hippo pathway. Our results exhibited reduction of YAP and enhanced DNA damage in the AMA placenta, as was observed in the skin and blood vessels of aged adults in other study. Prior to our study, YAP has been reported to decline in those placenta trophoblasts from pregnancies complicated by PE and IUGR (Liao et al., 2022). Not surprisingly, PE and IUGR placentas also showed a senescent phenotype. These facts implied that YAP expression possessed critical relevance in the placenta aging. DNA damage is a commonality during the development of the abnormal senescence phenotype (Lopez-Otin et al., 2023), which is determined by either endogenous or exogenous factors. Among the endogenous causes, oxidative stress is the dominant reason of injury. It has been realized that with increasing age, the accumulation of oxidized substances in the cells of the body tissue increases (Bruno et al., 2017; Sasaki et al., 2019), and the cells will suffer from oxidative damage. With these circumstances, there is apparent relationship among aging, YAP and oxidative damage. In fact, YAP transcription could be activated by nuclear (factor erythroid 2)-like 2 (NRF-2) for antioxidant defense and resisting senescence (Wu et al., 2013). Yet, the understanding of this relationship in placenta trophoblast, particularly in the context of AMA, is very limited.

As was reported in previous studies, oxidative stress contributed the senescence phenotype in species of cells (Parrinello et al., 2003; Chen et al., 2004), with no exception in HTR8/SVneo cell through our study. DNA damage induced by oxidative stress activated tumor suppressor protein p53 and its downstream p21 before SA-β-Gal staining getting positive. It was nothing strange that p53, along with p21, as important regulatory proteins of the cell cycle (Campisi and d'Adda di Fagagna, 2007; Coppe et al., 2008), responsed rapidly as self-protective mechanism once cells suffered adverse stimulation, to prevent the continued delivery of damaged genetic material, so their response preceded other senescence markers and persisted in senescent cells. Once activated, p53 exerts its functions in stimulating the transcription of apoptosis-related genes, thereby activating the apoptotic cascade, or activating p21 transcription to inhibit cells at G1 phase and repairing DNA damage (Ljungman, 2000). p16 can maintain cell cycle homeostasis and prevent hyper proliferation as placenta developed. It mediates senescence via the retinoblastoma (Rb) pathway by inhibiting the activity of cyclin-dependent kinases, ultimately resulting in G1 cell cycle arrest (Kuilman et al., 2010). As cells encounter stimulation, they may undergo senescence by activating either the p53-p21 or p16 pathway. If senescence is triggered by the activation of the p53-p21 pathway, cells had the potential to re-enter cell cycle once p53 inhibition lifted. Conversely, cells that undergo senescence exclusively through the p16-pRB pathway were unable to resume proliferation, even after inhibition of p53, pRB, or p16 (Beausejour et al., 2003). In this experiment, p16 did not exhibit a clear trend of change, which may be related to the stimulation and the duration of H_2_O_2_ exposure.

Interestingly, YAP of HTR8/SVneo cells showed a conspicuous decline soon after the oxidation challenge. Since YAP promoted cell proliferation, it was understandable that it attenuated as the correspondence to harmful stimulation, thus blocking the transmission of impaired genetic material. Our findings were consistent with a report that the oxidative stress inhibited YAP and downregulated eIF2αP-ATF4 signaling, resulting cell senescence and death in fibrosarcoma cell (Wang et al., 2024; Wu et al., 2015), implying that oxidative stress was the hazards factor of YAP decline. Besides, we found that the intracellular reactive oxidative species (ROS) (Supplementary Figure S6) and cell apoptosis (Supplementary Figure S7) existed even several days after the H_2_O_2_ attack, which illustrated the harmful effect would not clear away as the harmful strike vanished, highlighting the importance of avoiding harmful stimuli during pregnancy.

To further investigate the effects of YAP, we modulated YAP expression in HTR8/SVneo cells, which were originally obtained from a human first-trimester placenta and immortalized through gene modification, and found that YAP deficiency promoted premature aging, as reflected by the positive SA-β-Gal staining, along with improved expression of p53, p21 and p16. It happened that there was a similar case that depletion of YAP accelerated the premature aging in nucleus pulposus cell through stimulation of p53/p21, and lysosomal activity (Xie et al., 2013). These facts prompted that DNA damage likely occurred inside the YAP-knockdown cells since senescence resulted from DNA damage. Indeed, we detected the 8-OHdG in these YAP-deficiency HTR8/SVneo cells, the enhanced fluorescence intensity of 8-OHdG implying the improvement of DNA oxidative damage. 8-OHdG is one of the predominant forms of oxidative DNA lesions, which often gets significantly higher in those placentas of pregnancy complication (Takagi et al., 2004; Fujimaki et al., 2011; Kimura et al., 2013), including IUGR and PE. Furthermore, YAP was reported to decline in these complications (Liao et al., 2022), which may be a reason why 8-OHdG rises in these complications, but it needed further research for a more reliable conclusion.

In this study, we found that sh-YAP HTR8/SVneo cells exhibit an impairment in invasion ability, and it has already been well established that trophoblast invasiveness is critical in spiral artery remodeling (Lyall et al., 2013), which is of particular importance for normal placentation. In agreed with this result, the decreased invasion ability of low-YAP trophoblasts is involved in the pathophysiology of preeclampsia. Similar conclusions have been confirmed in another studies, indicating that reduced YAP levels may affect placental development by regulating trophoblast invasion and apoptosis, promoting pregnancy complications (Sun et al., 2018). Overexpression of YAP enhances the invasion of BeWo cells, HTR-8/SVneo cells, and JAR cells (Liu et al., 2020), which involves the interplay among Notch and WNT signaling pathways and YAP (Lin et al., 2023). Additionally, YAP is also regulated by microRNAs. For instance, miR-326 has been documented to target PAX8, leading to inhibition of YAP/TAZ-mediated transcriptional activity and consequently suppressing trophoblast proliferation, invasion, and migration (Zang et al., 2022). These facts distinctly suggested that besides for its role in regulating aging, YAP also positively regulates trophoblast biological functions.

It was deeply interesting that whether addition of YAP could attenuate DNA damage and ameliorate the senescence phenotype. In our study, the extra supplement of YAP at least partly compromised the senescence of HTR8/SVneo cells after oxidative stress strike, presented by the diminution in SA-β-Gal staining and decline of p53 expression. In fact, it has been reported that activation of YAP could promote nucleotide metabolism and decrease senescent phenotype (Santinon et al., 2018). Accordingly, 8-OHdG in the cells diminished, with the meaning of improvement of DNA oxidative damage, which implied that YAP played an essential role in antioxidant defense. In correspondence with our results, some antioxidant genes, containing MnSOD and catalase, can be upregulated by YAP-mediated FOXO1 activation in cardiomyocytes (Shao et al., 2014). We observed that p21 did not behave similarly as p53, which may be because of other involved p21 regulation factors such as growth factors, cytokines, or glucocorticoids (Londero et al., 2016). The specific mechanism that YAP ameliorated the DNA oxidative damage and senescence was still unclear, but at least it did not work by depressing ROS (Supplementary Figure S8).

YAP occupies a pivotal position in numerous biological processes within organisms, including but not limited to immune regulation, cell stemness and organ size control. To explore the underlying mechanisms by which YAP deficiency induces placental trophoblast senescence, RNA sequencing was performed. We conducted GSEA analysis on our RNA-seq data. The results revealed numerous DEGs in HTR8/SVneo cells that are potentially regulated by YAP and multiple DNA damage related pathways were enriched, including the DNA damage telomere stress induced senescence, DNA double-strand break pathways and inhibition of DNA recombination at telomere. Notably, many of these DEGs are related to histone expression, which are components of nucleosomes and play crucial roles in transcription regulation, DNA repair, DNA replication, and chromosomal stability (Liu et al., 2023). This implies that YAP participants in maintaining histone function, stability of DNA and chromosomal. In fact, histones and their post-translational modifications can regulate cellular senescence. Phosphorylated H3T11 histone alleviates vascular endothelial cell senescence, whereas lactylated H4 histone accelerates smooth muscle cell senescence (Wu et al., 2023; Li et al., 2024; Dubey et al., 2024). It is noteworthy that DNA damage serves as a common pathway in stress-induced senescence (Lopez-Otin et al., 2023). And several signaling pathways related to DNA damage-induced senescence were enriched in our RNA-seq analysis. Therefore, YAP potentially modulates the damage response of genetic material through its regulation of histones, ultimately triggering senescence phenotypes. However, further research is required to fully comprehend the regulatory interactions among YAP, histones, and DNA damage.

As essential transcriptional co-activator, YAP transcends mere regulation of placental senescence. The intricate equilibrium between the proliferation and differentiation of trophoblast is indispensable for placental development (Xiao et al., 2020). In the early stages of human placenta, the YAP-TEAD4 complex holds a central position in activating genes linked to cell cycle and stemness (Lin et al., 2023). Simultaneously, they suppress genes associated with cell fusion, facilitating the expansion of cytotrophoblasts (CTB) (Meinhardt et al., 2020). YAP functions as key regulator of cell proliferation, survival, placental differentiation, and migration. Although not mention the role of YAP in regulating trophoblast senescence in above studies, it is not difficult to find that once YAP is impaired, trophoblast cells exhibit typical characteristics of senescence, such as impaired cell proliferation, decreased cellular stemness, and compromised invasive capability (cell cycle arrest, weakened cellular stemness, and functional decline). So these functions of YAP are compatible with its ability in regulating senescence.

Besides, YAP take important part in preserving nuclear envelope integrity, partly by controlling the transcription of lamin B1 and ACTR2 directly (Sladitschek-Martens et al., 2022), which is indispensable for the construction of peri-nuclear actin cap (Vergnes et al., 2004). The actin cap surrounds the top surface of the nucleus, keeping the smooth expansion of the nuclear envelope and avoiding its distortion (Khatau et al., 2009; Kim et al., 2017). The integrity of nuclear envelope is crucial for protecting DNA from damage (Perez-Hernandez et al., 2022). In other words, impaired YAP disrupts the integrity of the nuclear envelope, rendering the DNA within the nucleus more vulnerable to oxidative damage from ROS. On the other hand, YAP may help resist oxidative stress through regulating antioxidant genes (Wu et al., 2013). Downregulation of YAP may lead to decreased synthesis of antioxidant enzymes, resulting in weakened ROS clearance and enhanced oxidative damage (Yeung et al., 2019). Thus, we hypothesize that the decline in YAP may be the cause of increased oxidative damage in AMA pregnancies. Other reports have referred to the possibility that lack of YAP may lead to adverse pregnancy outcomes by increasing oxidative damage. Bisphenol A (BPA) exposure during pregnancy would diminish YAP in trophoblast, causing damage to these cells and ultimately contributing to fetal growth restriction (Sun et al., 2024). And maternal vitamin D deficiency was associated with placental hypoplasia and intrauterine growth restriction, which may also be linked to reduced YAP levels (Wang et al., 2022). Consequently, pregnant women should exercise caution to avoid exposure to harmful substances like BPA and ensure adequate vitamin D intake to maintain optimal YAP levels, thereby lowering the risk of potential adverse pregnancy outcomes. This was particularly important for AMA pregnancy.

In this study, a significant senescence phenotype in term placentas from AMA pregnancy was accompanied by decreased YAP. In other study, YAP was found declining in skin and blood vessels with age (Sladitschek-Martens et al., 2022). As the placenta develops and matures, it is reasonable to assume that the importance of YAP’s role in maintaining stemness and promoting invasion diminish, resulting in its decrease. Concurrently, other genes that promote cell fusion and senescence become activated. That is to say, the decline in YAP may be a consequence of placental senescence. But the relationship between YAP and placenta senescence is seriously complex, as it can be caused by various factors, including maternal age, health status, lifestyle, and underlying diseases.

Nevertheless, our study is not without limitations. The HTR8/SVneo cell line, derived from first-trimester human placenta and immortalized with Simian Virus 40 large T antigen cDNA. Though it is used in studies of extravillous trophoblast (EVT) (Su et al., 2019) and part of trophoblast senescence (Xiong et al., 2021), it does not fully share the characteristics of all primary trophoblast, especially for those from term placenta. This was a non-negligible limitation in our work. We emphasize that the use of HTR8/SVneo cells as a model system provides valuable insights, but these findings should be interpreted with caution when extrapolated to *in vivo* conditions in placenta tissue near term. In future research, we aim to explore cell models that better mimic term placental trophoblast for more accurate and comprehensive findings.

In conclusion, our study preliminarily demonstrates that placental aging in AMA preganancy is associated with decreased YAP levels and increased oxidative damage, revealing the important role of YAP in protecting placental trophoblast from oxidative damage. Additionally, it suggests that YAP may serve as a potential intervention target for improving placental development and perinatal outcomes in AMA pregnancy. Nonetheless, there are limitations in this study, and further in-depth research is still required.

## Data Availability

The original contributions presented in the study are included in the article/Supplementary Material, further inquiries can be directed to the corresponding author.
