# Supplementary material for: Placental trophoblast aging in advanced maternal age is related to increased oxidative damage and decreased YAP
Source: Front Cell Dev Biol. 2025 Jan 21;13:1479960. doi: 10.3389/fcell.2025.1479960 (PMC11790555; doi:10.3389/fcell.2025.1479960)
Supplement: Supplementary file 2 [file DataSheet1.docx]

**Supplementary Data**


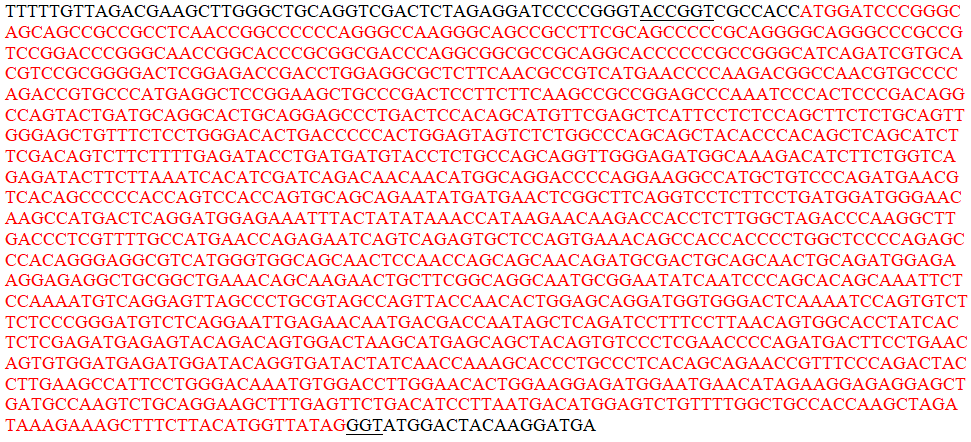


**Figure S1** Detailed sequences of the YAP-overexpression plasmid.

Black fonts, vector sequences; Underlined fonts, restriction enzyme cutting sites; Red fonts, insertion sequences.


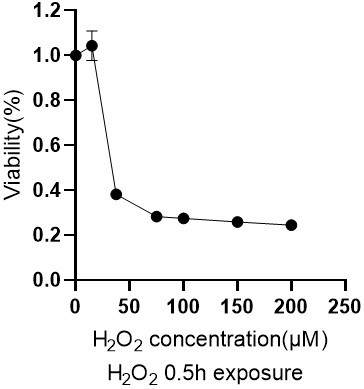

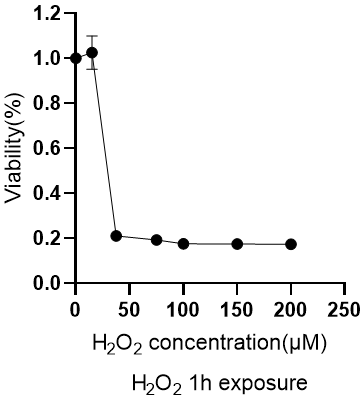

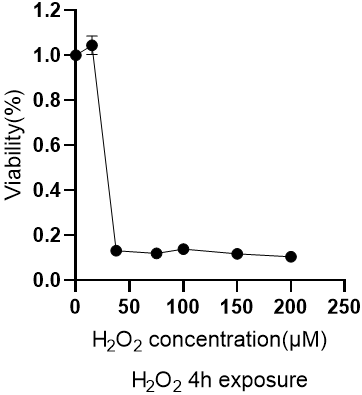

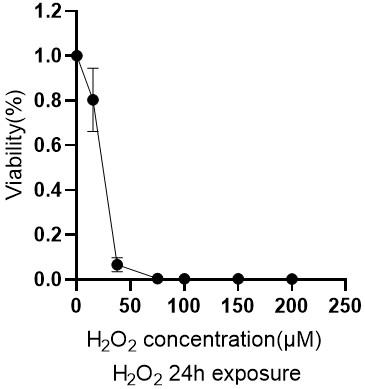

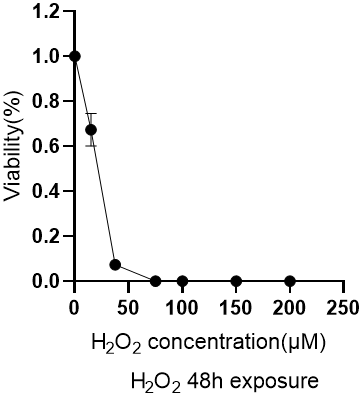


**Figure S2** The viability of HTR8/SVneo cells detected by CCK-8 assay.

The viability (%) of HTR8/SVneo cells after treated with H_2_O_2_ at the concentrations of 0, 15, 37.5, 75, 100, 150, 200 µM for 0.5, 1, 4, 24, 48 hours.


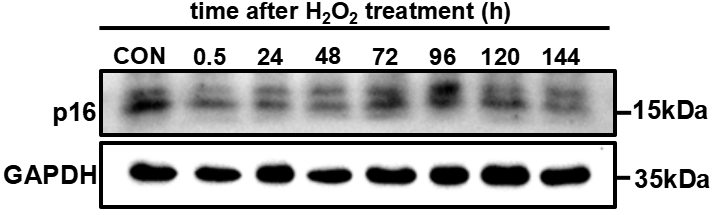

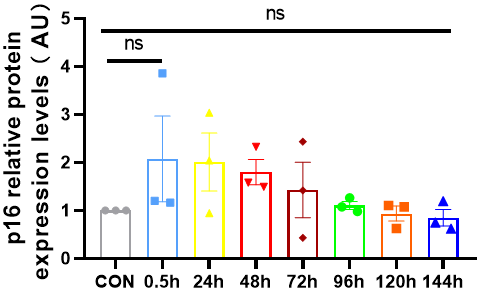


**Figure S3** Western blotting of p16 expression in HTR8/SVneo cells.

Western blotting of p16 protein expression in HTR8/SVneo cells over time after treated with H_2_O_2_ (n = 3). All data are presented as the mean ± SEM. ns: no significance. Student's t test and one-way ANOVA. AU, arbitrary unit.


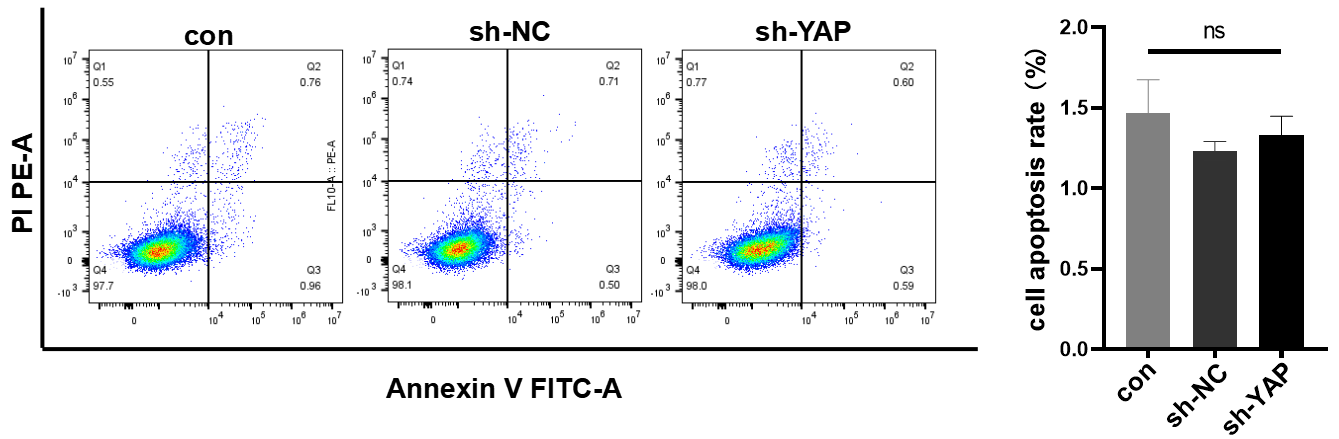


**Figure S4** Apoptosis of different HTR-8/SVneo cells detected by flow cytometry.

The apoptpsis rate (%) of different HTR-8/SVneo cells after treated with H_2_O_2_. sh-NC, negative control cells transfected with scramble shRNA; sh-YAP, cells transfected with shRNAs targeting YAP. All data are presented as the mean ± SEM. ns: no significance. One-way ANOVA.


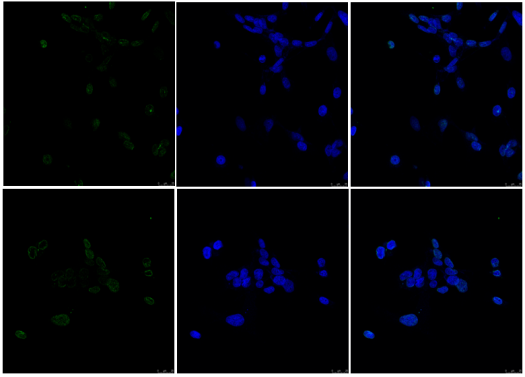


8-OHdG

DAPI

Merge

OE-NC

OE-YAP


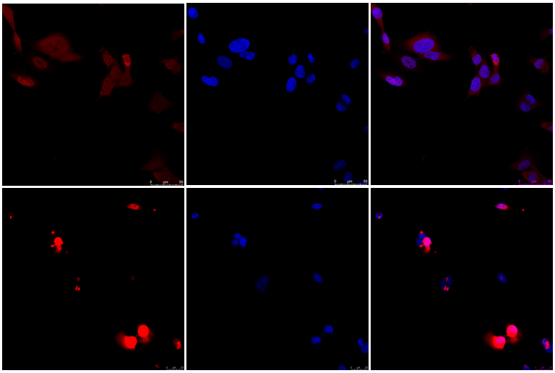


OE-NC

OE-YAP

YAP

DAPI

Merge

**Figure S5** IF staining in different HTR-8/SVneo cells.

IF staining of YAP (red) and 8-OHdG (green) in HTR8/SVneo cells; nuclei were counterstained with DAPI (blue). Scale bar, 100 μm.


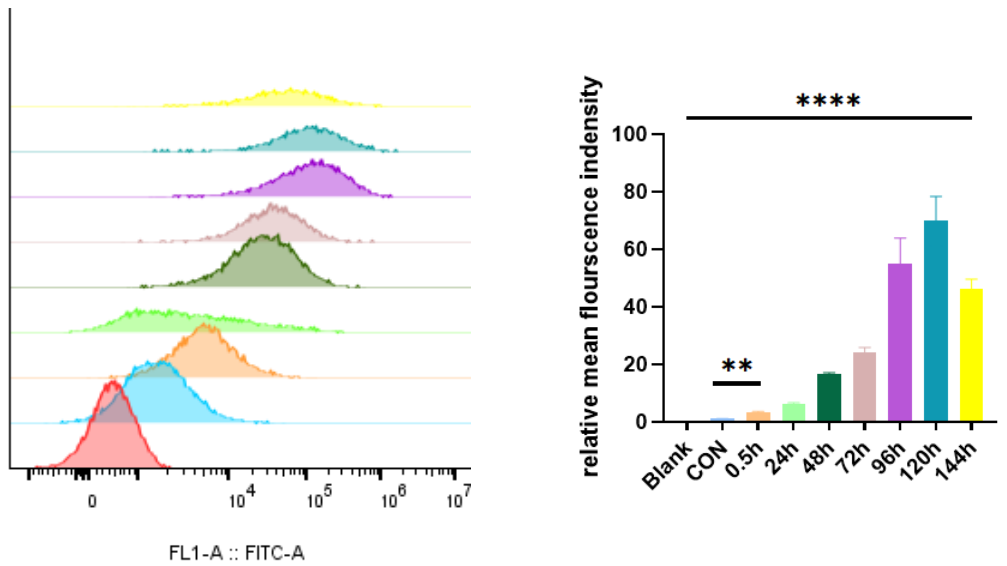


**Figure S6** ROS of HTR-8/SVneo cells detected by flow cytometry.

The ROS of HTR-8/SVneo cells over time after treated with H_2_O_2_. All data are presented as the mean ± SEM. ****p < 0.0001. Student's t test and one-way ANOVA.


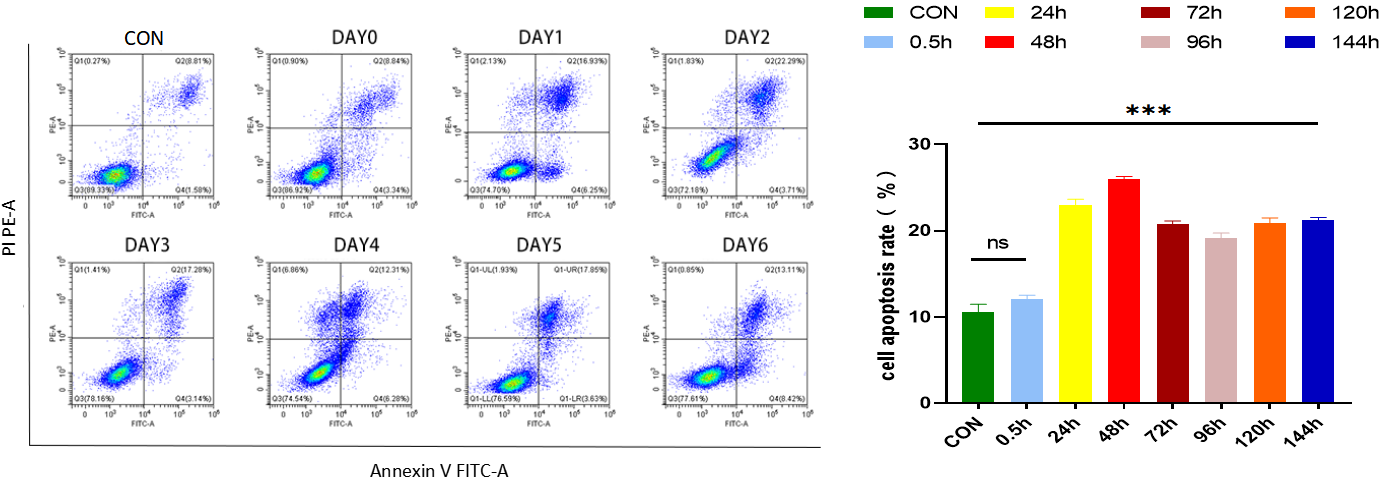


**Figure S7** Apoptosis of HTR-8/SVneo cells detected by flow cytometry.

The apoptpsis rate (%) of HTR-8/SVneo cells over time after treated with H_2_O_2_. All data are presented as the mean ± SEM. ***p < 0.001, ns: no significance.Student's t test and one-way ANOVA.


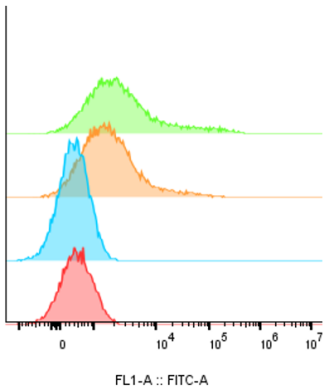

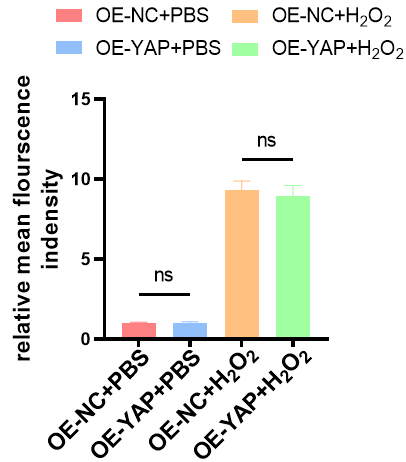


**Figure S8** ROS of different HTR-8/SVneo cells detected by flow cytometry.

The ROS of different HTR-8/SVneo cells after treated with H_2_O_2_. OE-NC+H_2_O_2_, negative control cells treated with H_2_O_2_; OE-YAP+H_2_O_2_, YAP overexpression cells treated with H_2_O_2_. All data are presented as the mean ± SEM. ns: no significance. Student's t test. AU, arbitrary unit.

**Supplementary methods**

**Plasmid amplification**

The competent cells (Biomed, Beijing, China) were placed on ice. Target plasmid was added to the EP tube containing competent cells according to the instructions ( gentle action and avoid creating bubbles) . The EP tube was then placed on ice for 30 min, then was placed for 42℃ metal bath heat shock for 45s, and the EP tube after heat shock was quickly placed on ice for 3 min. Add bacterial culture medium without antibiotics to the tube, gently mix and place the EP tube in the bacterial incubator (37℃, 200 rpm) for 1 hour to fully revive the bacteria.The EP tube was removed from the bacterial incubator, centrifuged at 3000rpm at room temperature for 3 min, and the bacterial culture medium was removed. The bacteria were resuspended in the remaining bacterial culture, and 10 μL of the bacterial suspension was absorbed onto a bacterial culture plate containing ampicillin, and then incubated in a biochemical incubator (37℃) for 16 hours before monoclonal colonies were formed. Single clones were picked into the 15 ml EP tube with bacterial culture medium containing ampicillin and incubated for 16 h (37℃, 200 rpm) .

**Plasmid extraction**

The bacterial solution was centrifuged for 10 min at 4000g at RT. According to the manufacturer's instructions of the Commercial kit (TIANGEN, Beijing, China) , discarded all media, added Solution I and blew the bacteria. Add Solution II, gently mixing down. Added N3 buffer and gently reversed several times. The lysed bacterial tube was placed into an ultra-fast centrifuge and centrifuged at 4℃ max speed (12000g) for 10 min. The supernatant was transferred to a new 2mL EP tube, and added equal volume ETR Solution, and gently reversed 10 times. According to the instructions, arranged the adsorption column and the elution tube, centrifuged at 10000 g for 1 min at RT, discard the waste liquid in the elution tube and put the adsorption column back into the elution tube. ETR elution buffer was added to the adsorption column, centrifuged at 10000g for 1 min at RT, and discarded. HBC buffer was added to the adsorption column, centrifuged at 10000g for 1 min at RT and discarded. Add elution buffer (diluted with absolute ethanol when noted), centrifuge at 10000g for 1 min at RT, discarded the waste and eluting once. The adsorption column was put back into the elution tube and emptied with the maximum speed (13000g) for 2 min at RT to remove the residual eluate. The adsorption column was placed in a new 1.5 mL EP tube, standing at RT for 5 min, and fully dried. ddH2O was added to the center of the column and centrifuged at 10000g for 1 min at RT. The DNA concentration was measured using the Nanodrop (NanoDrop Technologies, San Diego, USA).

**Lentiviral acquisition**

In order to obtain viruses containing the corresponding genes, the corresponding plasmids were transfected into HEK293T cells with jetPRIME® reagent (Cod. 101000046, Polyplus-transfection, Noisy-le-Grand, France) according to the manufacturer's instructions. Briefly, for optimal DNA transfection conditions, the cells were seeded in 10-cm dishes, and they were transfected at 80% confluent. First dilute 20 µg plasmid (target: psPAX2: pMD2G = 2: 1: 1) into 1 mL jetPRIME® buffer. Mix by vortexing. Then add 80 µL jetPRIME®, vortex for 1 sec, spin down briefly after vortexing jetPRIME® reagent. Incubate for 10 min at RT. Add 1 mL of transfection mix per well dropwise onto the cells in serum containing medium, and distribute evenly. After that gently rock the plates back and forth and from side to side, incubate the plate at 37 ℃. Replace transfection medium by cell growth medium 6 h after transfection. Cell culture medium supernatant (virus solution) was collected at 48 hours after transfection, and cell debris was filtered using a 0.45 µm filter.

**Lentivirus transfection**

HTR8/ SVneo cells were seeded at a number of 5 × 10^5^ in 6-wells plates. And each well of cells was transfected with 1 ml virus solution in the presence of polybrene (Solarbio, Beijing, China) , a infection enhancer according to the manufacturer's instructions. 24 h after transfection, the culture medium were replaced by fresh medium containing puromycin (1 μg/ml) for the selection of stable clones. Cells that are successfully infected could survive under the condition of puromycin, while uninfected cells would perish upon the puromycin.
